# Development of a Person‐Centered Measure of Dementia Caregiving Style

**DOI:** 10.1002/alz.084603

**Published:** 2025-01-09

**Authors:** Amanda N Leggett, Sophia Tsuker, Jennifer A Miner, Jin‐Shei Lai, Jonathan Troost, Noelle E Carlozzi

**Affiliations:** ^1^ Wayne State University, Detroit, MI USA; ^2^ University of Michigan, Ann Arbor, MI USA; ^3^ Northwestern University, Chicago, IL USA

## Abstract

**Background:**

Approaches to caregiving interventions are often “one‐size‐fits‐all”, yet family caregivers for individuals with dementia have unique caregiving styles with which they enact daily care. Mixed‐methods work by this team identified 5 distinct caregiving style profiles that vary in: orientation toward oneself or the care partner, adaptability, understanding of dementia, emotional expression, and behavioral management. This study seeks to develop a person‐centered assessment of caregiving style such that interventions and services can be targeted to caregivers’ unique styles of care.

**Method:**

Person‐centeredness of the Style measure is assessed with the NIH‐funded LINC‐AD’s Person‐Centered Measure Evaluation Tool (PC‐Met). Development phases assessed included: mixed‐methods exploratory research on caregiving style, iterative development and refinement of an item pool, cognitive interviews with caregivers, expert review, literacy and translatability review, and field testing of the refined items in 200 family/friend caregivers for a person living with dementia.

**Result:**

Person‐centered practices in the development of a caregiving style measure include: co‐creation (e.g., caregiver feedback throughout, caregiver interview), accommodation (e.g., full disclosure of topics, exploring across stage of disease), pragmatism (e.g., translatability review), incorporation (e.g., using positive versus loss language), biopsychosocial/cultural components (e.g., utility across multiple settings), and systemic focus (e.g., use of shared language).

**Conclusion:**

Caregiving style is associated with key outcomes of care (e.g., caregiver burden) and thus a person‐centered assessment measure can help tailor services and interventions to best fit unique styles of care, ultimately improving well‐being of the caregiver and quality of care for the person living with dementia.

